# Transforaminal lumbar interbody fusion combined with fat decompression for grade III idiopathic spinal epidural lipomatosis: a case report

**DOI:** 10.3389/fsurg.2026.1787460

**Published:** 2026-07-30

**Authors:** Xingsheng Yu, Hao Xing, Zhengqi Chang

**Affiliations:** Department of Orthopaedics, The 960th Hospital of People's Liberation Army of China (PLA), Jinan, China

**Keywords:** case reports, lumbar spinal stenosis, spinal decompression surgery, spinal epidural lipomatosis, transforaminal lumbar interbody fusion

## Abstract

**Background:**

Spinal epidural lipomatosis is a rare condition characterized by the accumulation of fatty tissue in the epidural space of the spinal canal, leading to compression of the spinal cord or nerve roots.

**Case description:**

A 72-year-old male patient presented to our hospital with a 6-month history of lower back pain, bilateral lower limb numbness, and difficulty in walking. After a comprehensive evaluation, the patient was diagnosed with Grade Ⅲ Idiopathic Spinal Epidural Lipomatosis. He then underwent Transforaminal Lumbar Interbody Fusion (TLIF) combined with fat decompression surgery. The patient's symptoms significantly improved postoperatively, and he had no significant discomfort or related complications during the 3-month postoperative follow-up visit.

**Conclusions:**

TLIF interbody fusion combined with fat decompression surgery is an effective procedure for patients with Grade Ⅲ idiopathic spinal epidural lipomatosis.

## 1 Introduction

Spinal Epidural Lipomatosis (SEL) is a rare condition characterized by abnormal accumulation of normal fat tissue outside the dura mater within the spinal canal, leading to lower back pain, intermittent claudication, and bilateral progressive lower limb numbness and weakness (1). The diagnosis of SEL mainly relies on imaging studies, with Magnetic Resonance Imaging (MRI) being the primary imaging modality. Borré et al. (2) classified SEL into four grades based on the thickness of epidural fat and the ratio of the anteroposterior diameter of the spinal canal on MRI imaging. Although the pathogenesis of SEL is currently unclear, it can be roughly divided into obesity type, steroid hormone intake type, and idiopathic type. In cases with acute neurological deterioration or those that are unresponsive to conservative treatments, surgical intervention is recommended. According to the literature, laminectomy or vertebral plate resection combined with epidural fat reduction is a common surgical procedure for SEL (3–7). We present the case of a patient with Grade Ⅲ Idiopathic Spinal Epidural Lipomatosis, along with a detailed report and analysis of the patient's general condition, clinical manifestations, diagnosis, and treatment process to improve current understanding of this rare disease.

## 2 Case report

**Figure 1:**
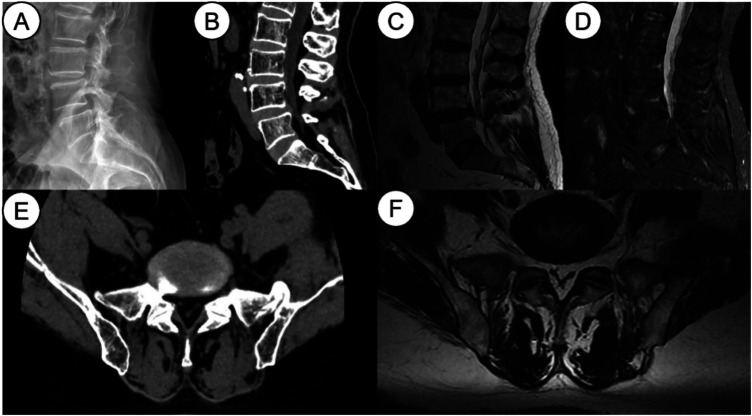
**(A–D)** Results of imaging examinations performed on the patient upon admission (lateral x-ray of the lumbar spine, lumbar spine CT, lumbar spine MRI T2, and T2 fat suppression sequences); **(E)** and **(F)** show the transverse CT and MRI T2 sequences of the L5-S1 segment, where nerve dura deformation due to fat compression can be observed, forming a “V”.

A 72-year-old male patient presented to our hospital with a 6-month history of lower back pain, bilateral lower limb numbness, and difficulty in walking. He had not received formal treatment prior to presenting at our facility; however, the patient had opted to administer oral pain medications and topical ointments at home with unsatisfactory results. He denied any history of steroid use or endocrine metabolic disorders, and his Body Mass Index (BMI) was 24.4 kg/m^2^. The patient had an intermittent claudication gait, normal lumbar lordosis, mild restriction in lumbar flexion and extension, evident tenderness and percussion pain in the lower lumbar spine radiating to both lower limbs, decreased sensation in the skin of both buttocks, outer sides of both lower limbs, dorsum and sole of both feet (more pronounced on the left side), and decreased sensation in the saddle area, with normal muscle strength and tone in both lower limbs. He also had a negative straight leg raising test bilaterally, normal cremasteric reflex, normal knee-jerk reflex, absent ankle-jerk reflex, and negative Babinski and Hoffmann signs bilaterally. The patient's lumbar spine x-ray and CT scan showed instability and mild anterior displacement of the L4 vertebra, as well as a low-density mass in the epidural space at the L5/S1 level. The CT values of the mass were consistent with fat density and was mainly located on the side and back, causing compression and displacement of the dural sac and nerve roots. The dural sac appeared to have a “V”-shaped change (Figures 1A–E). MRI revealed disc herniation at the L4/5 level, resulting in compression of the corresponding dural sac. The abnormal signal in the epidural space at the L5/S1 level appeared as a high signal on T1WI and medium-high signal on T2WI, which is suppressed on fat-suppressed sequences, indicating the accumulation of fat tissue around the dural sac, leading to the “V”-shaped change of the dural sac (Figures 1C–F; Figures 3A,B).

**Figure 2:**
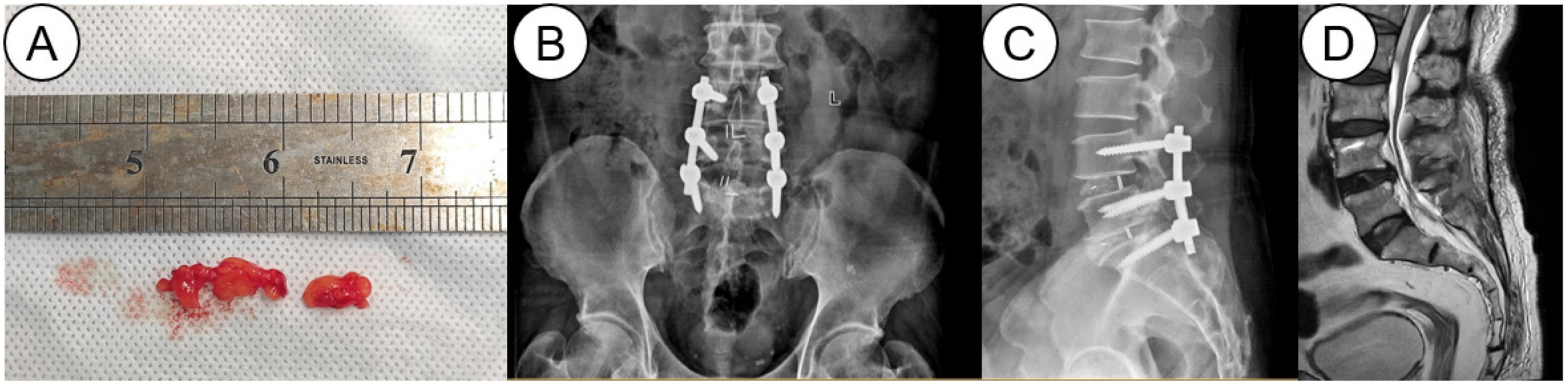
**(A)** Fat tissue obtained intraoperatively from the spinal canal; **(B)** and **(C)** display postoperative lateral and anteroposterior x-rays of the lumbar spine fixation; and **(D)** depicts a postoperative lumbar spine MRI T2 sequence.

Based on the patient's diagnostic imaging and clinical results, the diagnosis of SEL combined with lumbar instability was confirmed. After ruling out surgical contraindications, the patient underwent L4-S1 segment TLIF interbody fusion combined with fat decompression surgery (Figures 2A–C). The surgery was performed by a senior spine surgeon. After successful administration of anesthesia, the patient was placed in the prone position with the abdomen slightly elevated, and routine disinfection and draping were performed. Under the guidance of a C-arm fluoroscope, the L4/5 and L5/S1 intervertebral spaces were located on the skin surface. The positions of the L4, L5, and S1 spinous processes were identified, and points were marked approximately 2 cm lateral to both sides of the spinous processes. A 10.0-cm incision was made 2.0 cm lateral to the midline spinous processes. The lumbar fascia was incised and explored, and soft tissues were adequately released around the vertebral plates. Using the C-arm fluoroscope for visualization, pedicle screws were inserted into the L4, L5, and S1 vertebral arches. Bilateral laminectomy and decompression at L5/S1 were performed extending medially to the midline and laterally to the outer border of the intervertebral foramen, removing posterior epidural fat tissue to thoroughly free the dura mater and nerve roots. After the complete removal of the nucleus pulposus and cartilaginous endplates, the operation site was rinsed with saline before the insertion of a fusion cage filled with bone grafts into each intervertebral space. The anterior part of the vertebral body was filled with bone graft and secured using a pedicle screw rod assembly with appropriate compression. Fluoroscopy confirmed optimal positioning of the internal fixation and fusion cages. The surgical site was then rinsed with saline after achieving hemostasis, and a drainage tube was inserted before closing the incision in layers.

One week postoperatively, the patient reported improvement in back pain with the VAS scores improving from 8 to 3; the lower limb pain VAS score also improved from 8 to 2. The patient's Oswestry Disability Index (ODI) improved from 70% to 20%, and their saddle area sensation function also improved compared to the preoperative state. No discomfort was reported during the 6-week and 3-month follow-up visits. The patient provided written informed consent to publish this case. This study was approved by the ethics committee of our hospital.

## 3 Discussion

SEL is a rare spinal condition in which normal fatty tissue accumulates in the epidural space, causing compression of the spinal cord or nerve roots. Its symptoms, such as back pain, numbness, lower limb pain, and cauda equina syndrome, are similar to those caused by lumbar disc herniation or lumbar spinal stenosis, making it easy to overlook (8). Thomas et al. (9) assessed 28,902 adults and found that approximately 1 in 40 patients exhibited signs of SEL on spinal MRI images. Among them, 23% were asymptomatic, 72% had spine-related symptoms, and 5% had symptoms specific to SEL. Several studies have shown that SEL is more common in the L5-S1 segment with a male preponderance (10, 11). Consistent with previous reports, the patient in this study was male with a lesion at the L5-S1 level combined with L4 vertebral instability. He complained of lower back pain, bilateral lower limb numbness, and difficulty in walking. Physical examination revealed significant tenderness and percussion pain in the lower lumbar spine, radiating to both legs. Sensory loss was noted in the buttocks and outer calves, as well as the dorsum and soles of the feet (more on the left side than the right), with reduced sensation in the saddle area. Previous studies have shown that when MRI indicates increased continuity of fat within the compressed area of the spinal canal and a fat anterior-posterior diameter > 7 mm, a diagnosis of SEL can be made (12). According to Borré et al. (2), using the ratio of the anterior-posterior diameter of the dural sac to the anterior-posterior diameter of the epidural fat, and the ratio of the anterior-posterior diameter of the epidural fat to the anterior-posterior diameter of the spinal canal, SEL can be classified into four levels of severity. This case falls under Borré's Grade III classification. Moshe et al. (13) proposed the following four causes of SEL: 1) exogenous intake of corticosteroids; 2) obesity; 3) endogenous increase in cortisol (Cushing's syndrome); and 4) idiopathic. The patient in this case had a BMI of 24.4 kg/m^2^, which falls within the normal weight range (18.5∼24.9 kg/m^2^) and had no history of concurrent exogenous or endogenous intake of corticosteroids; therefore, he was considered to have Grade III idiopathic SEL.

**Figure 3:**
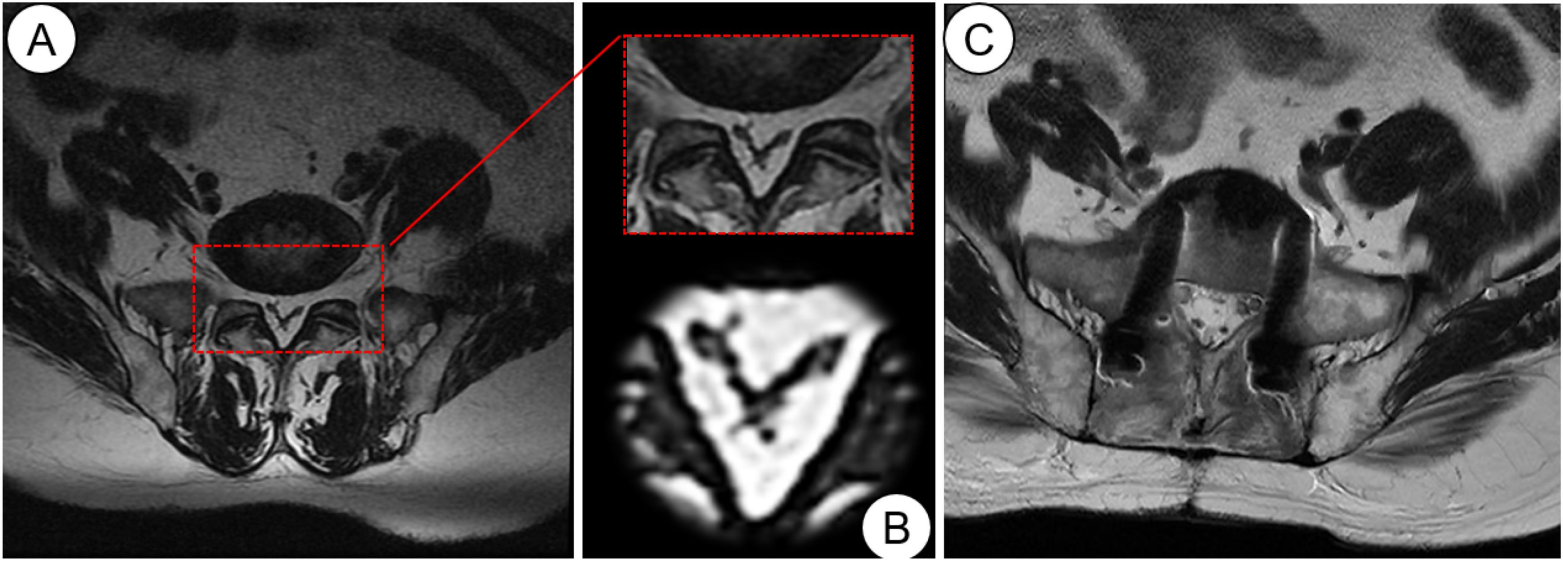
**(A)** accumulation of fat within the lumbar spinal canal on lumbar spine MRI T2; **(B)** is an enlarged view of panel **(A)**, showing a “V” shaped compression of the dura mater; **(C)** postoperative lumbar spine MRI indicating the compressed dura mater has returned to its original shape.

SEL has atypical clinical manifestations that need to be differentiated from various diseases such as epidural cysts or hemorrhage, vertebral metastases, and paraspinal lesions. However, it has characteristic imaging findings, with increased intraspinal fatty tissue surrounding the entire dural sac. In the lumbar spine, adipose tissues predominantly accumulate on the dorsal side of the dural sac, compressing it towards the ventral side and producing a triangular shape on cross-sectional imaging. In the lumbosacral region, the fatty tissue surrounds the dural sac and compresses it into a star shape, with three bands radiating from the center resembling the letter “Y” on imaging, and is often referred to as the “Y” sign (14). The patient in this case is classified as Borré Grade III SEL, with significant epidural fat occupying the ventral and bilateral dorsal sides of the lumbosacral dura, causing compression and forming a “V” sign (Figures 3A,B). Furthermore, the CT image showed a triangular low-density area on the dorsal side of the dural sac on cross-sections, while a lumbar spine MRI revealed compression of the involved segments of the dural sac and increased fatty tissue outside the dural sac. In MRI sequences of SEL, the fat signal in the T2 and T2WI fat-suppressed images is significantly suppressed (12).

Currently, the treatment options for SEL are either conservative or surgical. Conservative treatment includes bed rest, pain relief, weight reduction, and discontinuation or reduction of the use of exogenous hormones. Valcarenghi et al. (15) reported the case of an obese 48-year-old male patient (BMI 37.4 kg/m^2^) with a history of chronic back pain who was diagnosed with idiopathic SEL. After undergoing sleeve gastrectomy, his patient's symptoms completely resolved after 6 months of follow-up. If a patient with SEL shows severe or progressive neurological symptoms and/or no improvement in clinical symptoms with conservative treatment, surgical treatment becomes necessary. The main goal of surgical treatment is decompression by removing the lamina and fat within the spinal canal. Various surgical strategies for SEL have been reported, including laminotomy, endoscopic-guided fat aspiration, microsurgical laminotomy or multilevel laminectomy, fat decompression surgery, and lateral fusion after internal fixation (3–7) (Table 1). Liu et al. (16) successfully treated three cases of symptomatic spinal epidural lipomatosis using UBE technology, proving it to be a minimally invasive muscle-sparing technique that achieves nerve decompression by removing excessive fat tissue accumulation within the spinal canal. However, the optimal efficacy of UBE technology is limited to specific cases, with its clinical application being constrained by various factors. For patients with severe spinal deformities, lumbar spondylolisthesis, or severe osteoporosis, there is a higher risk of iatrogenic injury with this technique, potentially leading to postoperative spinal instability due to insufficient vertebral stability. In complex cases with extensive lesions, such as multi-level spinal canal fat accumulation or Grade III spinal epidural lipomatosis, pure UBE decompression may not completely remove the lesions, necessitating combined open surgery or other procedures. Patients with spinal epidural lipomatosis often have concurrent lumbar degenerative changes, such as disc degeneration, facet hypertrophy, and vertebral body thickening, leading to decreased spinal stability. Decompression surgery alone (including UBE) requires the removal of some bony structures like lamina and facet joints to achieve nerve release, further compromising the spinal “three-column structure” (especially the posterior column), significantly increasing the risk of postoperative spinal instability. Utilizing pedicle screw fixation + interbody fusion (such as PEEK Cage fusion) can securely connect the affected segment with adjacent vertebrae, forming a rigid stable structure, completely eliminating abnormal movement. For patients with osteoporosis or severe spondylolisthesis associated with spinal epidural lipomatosis, fusion surgery is crucial in preventing postoperative spinal collapse.

**Table 1:** Reported management of spinal epidural lipomatosis in recent literature.

Author	Year	Age	Gender	Part	Grade	Treatment	Remarks
Fabrice Mallard (19)	2021	51	F	L4-L5 L5-S1	III	L4-S1 posterior spinal decompression and L4-L5 posterior spinal instrumented fusion	/
Yusuke Shimada (20)	2023	87	M	L2-L3	/	Posterior lumbar decompression surgery	Diagnosed with microscopic polyangiitis and treated with prednisone at a 5 mg daily dose
Xiao Shenmao (21)	2022	55	F	L4-L5	III	Lumbar intervertebral disc and partial laminectomy, intervertebral bone graft fusion internal fixation and intraspinal fat removal	History of rheumatic heart disease for 5 years, autoimmune hepatitis for 12 years, taking hydrochlorothiazide tablets, prednisone acetate tablets; diagnosed with diabetes for 4 months, self-injecting insulin.
Ayman A. Salman (12)	2024	17	M	T6- T10	/	T6-T10 transpedicular fixation for correction of kyphosis, debulking of the spinal epidural fatty tissue, and left hemilaminectomy for biopsy	BMI = 25 kg/m2
Yahao Li (18)	2025	37	F	L2-L3	/	Unilateral biportal endoscopy	BMI=30.48 kg/m2
Papastefan ST (22)	2018	9	F	C2-C6	/	Underwent C2-C7 laminoplasty for resection of the epidural lesion	/
Valcarenghi J (15)	2018	48	M	L4-L5	/	sleeve gastroplasty	BMI=37.4 kg/m2
Han X (7)	2021	53	M	L2-L5	II	Intraspinal decompression, modified articular fusion and adipose resection	BMI=33.3 kg/m2
Yang K (8)	2021	55	M	L3-L5	III	Fat debulking, lamina osteotomy and replantation *in situ*, and titanium plate fixation	/
Kang S-S (23)	2019	62,78,46	1M/2F	L4-S1，L2-S1,L4-S1	II, II, III	Percutaneous biportal endoscopic surgery	/
Frank E (5)	1997	53	M	L5-S1	/	A small bilateral L5-S1 laminotomy was performed and copious epidural fat was encountered. The majority of the fat was removed easily using endoscopically guided malleable suction apparatus	/

The “/” symbol indicates a lack of information; M, male; F, female; L, lumbar vertebrae; S, Sacral vertebrae; BMI, body mass index.

In this case, due to instability in the L4 vertebra and severe compression of the dural sac's anterior and posterior fat in the L5/S1 segment, minimally invasive techniques were unable to fully address the two causes of our patient's discomfort. Hence, the patient underwent a lumbar vertebral interbody fusion from the L4 to S1 segments due to L4 vertebral instability. During the procedure, part of the vertebral plate and intervertebral disc were removed, along with the decompression of fat tissue causing nerve compression within the spinal canal, achieving the desired decompression effect. Postoperatively, the nerve dura compressed by fat tissue regained its shape (Figure 2D; Figure 3C). Zhang et al. (17) suggested that vertebral plate resection was suitable for severe nerve defects, as removing excess fat can effectively alleviate nerve compression. The patient in this case had severe dural compression at the L5/S1 segment but did not present with symptoms suggestive of severe nerve damage, such as those of cauda equina syndrome, including urinary or fecal incontinence. Therefore, we only performed TLIF interbody fusion surgery combined with fat decompression, and the patient's nervous symptoms improved postoperatively. Pengfei Yu et al. (18) decompressed the spinal canal of a 37-year-old female patient with symptomatic SEL using UBE technology and achieved good surgical outcomes. They reported that UBE may be an effective alternative to open surgery for treating patients with symptomatic SEL, with the advantages of minimal trauma and fast recovery. However, due to the advanced age of this patient and concurrent L4 vertebral instability, UBE technology was not an optimal treatment choice.

This study has two main core limitations: firstly, it is only a single case report without controls, lacking support from cohort data, thus having limited evidence grade, making the conclusions not generalizable to all patients with type III thoracic spinal epidural lipomatosis; secondly, the follow-up duration is only 3 months, constituting a short-term observation that cannot assess long-term adverse events such as recurrence of spinal epidural fat hyperplasia, loss of spinal stability, and recurrence of neurological symptoms. The long-term effectiveness and safety of this procedure still require an extended follow-up period for further validation. However, our report shows that TLIF surgery can effectively improve patients' quality of life within 3 months postoperatively and is expected to become an effective treatment for such complex diseases. In conclusion, for patients with degenerative spondylolisthesis, segmental instability, severe multilevel disc degeneration, advanced age-related osteoporosis, and diffuse SEL grade III-IV, TLIF surgery (decompression with fusion) can alleviate nerve compression caused by adipose tissue.

## Data Availability

The datasets presented in this study can be found in online repositories. The names of the repository/repositories and accession number(s) can be found in the article/Supplementary Material.
